# Enhancing Drafter Performance in Spunbonding of Polymeric Fibers via Airflow Simulation

**DOI:** 10.3390/polym18020187

**Published:** 2026-01-09

**Authors:** Behrang Mohajer, Mohamad Kheradmandkeysomi, Chul B. Park, Markus Bussmann

**Affiliations:** Microcellular Plastics Manufacturing Laboratory (MPML), Department of Mechanical and Industrial Engineering, University of Toronto, Toronto, ON M5S 3G8, Canada; bmohajer@mie.utoronto.ca (B.M.); mohamadk@mie.utoronto.ca (M.K.)

**Keywords:** spunbonding, polymer fibers, fiber drawing, drafter design, nonwovens, computational fluid dynamics (CFD), process optimization, airflow simulation

## Abstract

Spunbonding drafters play a decisive role in determining fiber attenuation, morphology, and final nonwoven quality; however, their internal airflow behavior remains poorly characterized due to limited physical accessibility and historically empirical design practices. This work employs high-fidelity computational fluid dynamics (CFD) to systematically resolve the airflow field inside a laboratory-scale drafter and to quantify the impact of geometry on fiber drawing conditions. The simulations reveal a previously unreported “braking effect,” where adverse flow structures reduce effective shear drag, limit drawability, and increase the likelihood of fiber breakage. Parametric virtual experimentation across seven geometric variables demonstrates that the drafter configuration strongly governs shear distribution, flow uniformity, and energy consumption. Using a performance-oriented optimization framework, three key processing objectives were targeted: (i) maximizing shear drag to promote stable fiber attenuation, (ii) improving axial drawing uniformity, and (iii) minimizing pressurized-air demand. CFD-guided design modifications—including controlled widening, tailored wall divergence and convergence, and an extensible lower section—were implemented and subsequently validated using a newly constructed prototype. Experimental measurements on polypropylene (PP) and high-density polyethylene (HDPE) fibers confirm substantial reductions in fiber breakage and improvements in drawing stability, thereby demonstrating the effectiveness of simulation-driven process optimization in spunbonding equipment design.

## 1. Introduction

Spunbonding [1,2] has for decades been a method of producing polymeric nonwoven textures for filtering masks, diapers, liquid absorbents, etc., on an industrial scale, and is a similar process to melt spinning (e.g., [3,4]) and melt-blowing (e.g., [5,6,7]). In particular, spunbonded products are thin and exhibit mechanical strength that makes them suitable for medical filtering, as used during the COVID-19 pandemic and for nano-fibrillation products (e.g., [8,9,10,11]). Spunbonding has attracted more attention recently (e.g., [12,13,14,15]), but there remains significant potential for its improvement, particularly from the perspective of polymer processing and fiber manufacturing efficiency.

**Figure 1:**
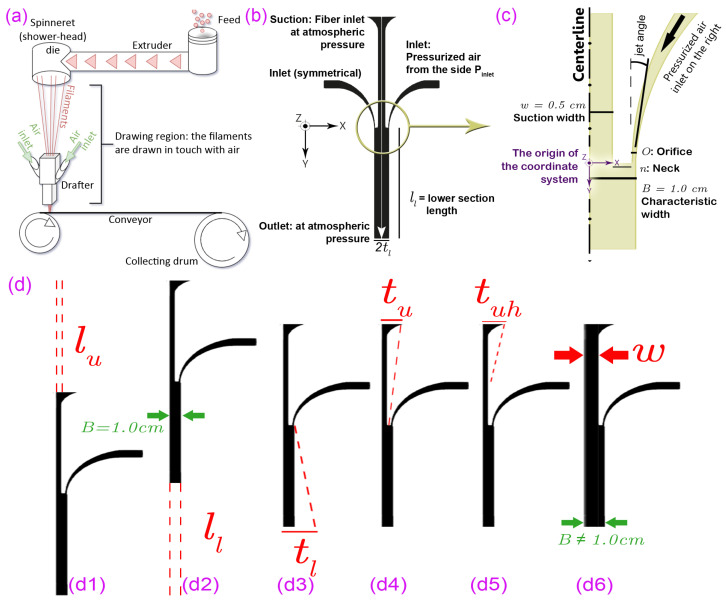
The schematics of the spunbonding process and the drafter. (**a**) Spunbonding components. (**b**) The 2D geometry of the drafter. The white arrow along the center represents the symmetry plane. That is the centerline where the fibers are drawn. (**c**) Zooming into the right half of the mixing section of the drafter. All fibers are drawn on the YZ plane parallel to each other. (**d**) Schematics of the geometrical design parameters discussed in this study. (**d1**) upper section length, $l_{u}$, (**d2**) lower section length, $l_{l}$, (**d3**) angle of the lower side wall, $t_{l}$, (**d4**) angle of the upper side wall, $t_{u}$, (**d5**) angle of the upper half of the upper wall, $t_{uh}$, and (**d6**) the width of the upper section, *w*. The characteristic width, *B*, is the overall width of the drafter’s lower section and changes only in case (**d6**).

In spunbonding, polymer pellets are melted and extruded through a spinneret die, forming thin, long fibers that elongate and crystallize as they move downwards along the drawing region (Figure 1a). These fibers thin down to a freezing point [16,17] at a certain vertical distance from the die and continue cooling as they pass through the drawing region and into a drafter. With potentially tens or hundreds of fibers involved, this extra length ensures that all fibers, especially those located closer to the center, solidify sufficiently before entering the drafter.

Gravity and shear drag ($F_{\mathrm{drag}}$, also known as skin friction) are the drawing forces and exert elongational stress on the melt, inducing crystallization within the fibers [18,19] and enhancing the mechanical strength of the fibers significantly [20]. Near the die, gravity predominates, but shear drag quickly becomes crucial for the rest of the drawing region and within the drafter. The drafter, a fixed device with two inlets and an outlet, plays a key role in providing the necessary shear drag for thinning the fibers (Figure 1b). Controlling this airflow–fiber interaction is therefore central to polymer fiber formation and the resulting nonwoven properties.

Precise control of shear drag is essential to address challenges such as fiber breakage and ensure sufficient drawing [21,22]. Moreover, controlling the amount of drawing force becomes crucial when the throughput material is subject to small changes and fluctuations, often related to factors like the quality of the feed, warm-up, and the distribution of additives. Traditionally, controlling the drafter inlet pressure achieves this, but changes in pressure can disrupt airflow uniformity, and literature on this is scarce.

The traditional approach to improving spunbonding processes has been through slow trial-and-error methods [23,24], with minimal changes to the spunbonding machine, which have remained largely unchanged for decades [25]. This conservatism arises from the challenges of modeling the complex multi-physics interactions at the fiber–air interface, compounded by the significant dependence of the process on material properties. Consequently, adjustments to crucial factors like shear drag are typically made experimentally. Despite these challenges, simulation studies have contributed to understanding the process, with recent efforts focusing on simplifying models to expedite analysis [26,27,28,29,30,31,32,33,34]. However, the geometric design of the drafter, a critical component in spunbonding processes, has received limited attention in the literature, despite its significant potential to impact process effectiveness. In particular, to our knowledge, no simulation studies have focused specifically on the internal airflow of the drafter and its design optimization, which we address in this work through CFD-based analysis of airflow patterns relevant to fiber drawing.

The drafter is a fixed device with three inlets (one on top and one on each side) and a single outlet at the bottom (Figure 1b). It works analogously to a suction pump: as the fibers fall due to their weight, two pressurized air jets flow into the drafter and accelerate the fibers through shear drag until they exit the outlet. Figure 1c illustrates the pressurized inlet entering from the orifice at Y=0 inside the drafter.

Excessive shear drag from the drafter may cause fiber breakage in two main areas: within the drafter, characterized by extreme velocity variations, or above the drafter, where the fibers are still in the molten phase. Our observations indicate that side fibers are more susceptible to breakage compared to those at the center.

Instead of comprehensive two-phase simulations, we focus on airflow using computational fluid dynamics (CFD) simulations to analyze the drawing forces involved and to improve drafter design for industrial spunbonding. This approach aligns with simulation-driven process optimization strategies commonly employed in polymer processing. Our simulation results aim to (a) maximize drawability [35,36], (b) shear drag uniformity, and (c) minimize energy consumption, while commenting on drafter modifications to achieve these goals.

We assess the air velocity (*U*) distribution within the drafter as it plays a crucial role in achieving goals (a) and (b). It directly influences the shear drag, which is proportional to ${\left(U-U_{\mathrm{fiber}}\right)}^{2}$. In our experimental setup, the polymer melt exits the die holes with a diameter of 0.4–0.6 mm at a velocity of $U_{\mathrm{fiber}}=1$–2cm/s, and under certain process conditions, it accelerates to the range of 20 to 40m/s. One challenge to achieving drawing uniformity is the significant variation in *U* inside the drafter, unlike the constant $U_{\mathrm{fiber}}$ below the freezing point. This difference complicates the task of ensuring a uniform downward shear drag. Lastly, we analyze the mass flow rates of the pressurized inlet air consumed in the drafter to assess goal (c).

In the following sections, we first present our simulation methodology, including the baseline geometry and the corresponding experimental setup. The resulting flow fields are analyzed and compared to experimental observations to evaluate the accuracy and applicability of the proposed simulation framework. This direct comparison with measured data provides essential experimental validation of the airflow patterns and supports the practical relevance of the design modifications investigated. We then describe a series of systematic design modifications introduced to simulate the airflow in the spunbonding process.

## 2. Materials & Methods

**Figure 2:**
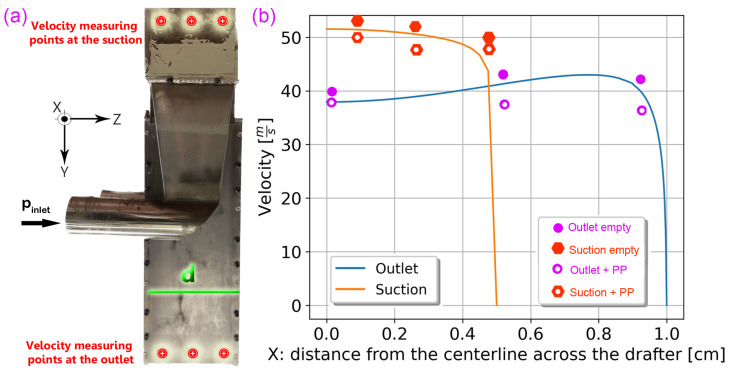
The experimental drafter. Many fibers are drawn in parallel along *d*, the depth of the drafter in the *Z* direction. (**a**) The airflow measuring points. (**b**) Velocity distribution across the outlet and the suction compared to the experimental data. The purple and red points correspond to the averages of our velocity measurements with and without PP fibers in the drafter.

We investigated several design parameters for the drafter, and in this paper, we discuss the ones that significantly improve the drawing process. These parameters include the inlet pressure, lengths, width, and side wall angles as listed in Table 1 and depicted in Figure 1d. We begin our discussion with a base geometry corresponding to that of our laboratory drafter (Figure 2a). Upon experimental validation of our simulation, detailed in Section 2.1, we systematically modify the selected design parameters, conducting comparative analyses of the resultant outcomes. Finally, we summarize our findings and present recommendations for modifying the drafter.

### 2.1. Experimental Setup

Air velocities were measured at two accessible locations of a custom-designed spunbonding machine [21,37], specifically at the suction and outlet of the drafter (Figure 2b), using an AVbase Dwyer anemometer/manometer (Dwyer Instruments, LLC, Michigan City, IN, USA). This approach was necessitated by the difficulty of accessing the internal region and the potential for the anemometer to disturb the internal air and fiber flows within the narrow channel of the drafter, thereby invalidating the measurements.

Measurements were conducted both in the absence and presence of polymer feed, namely general-purpose polypropylene (PP) H521 from Braskem America, Inc. (Philadelphia, PA, USA) and ASPUN™ 6835A Fiber Grade Resin high-density polyethylene (HDPE), The Dow Chemical Company (Midland, MI, USA). These polymers were chosen for their favorable spinnability and low cost, facilitating reproducibility and enabling further research in future studies.

Figure 2a presents the side view of our drafter and the specific locations at which the air velocity was measured. Figure 2b shows the corresponding experimental measurements obtained by suction at the outlet boundaries, both with and without fibers, resulting in six scattered data points. For comparison, the simulated continuous velocity profiles are also included to demonstrate (1) the reasonable agreement between the simulations and the measurements, and (2) the negligible influence of fiber on the airflow within the drafter.

The extruder consisted of four heating zones with set temperatures of 180, 190, 200, 200, and 200 °C, while the die temperature was maintained at 220 °C for both materials. All experiments were performed on a laboratory-scale base geometry equipped with 90 spinneret holes, each with a diameter of 600 μm.

Fiber diameters were measured from collected samples using a 1600× Wendry Digital Electron Microscope, model B0813J4TKF, manufactured in Shenzhen, Guangdong, China. The diameters were quantified with ImageJ [38] (Version 1.54p) using a 10 μm calibration slide, and the average values were reported. Additional details on the fiber-diameter measurements for the new drafter design are provided in Appendix C.

### 2.2. Computational Methodology

A CFD model was developed based on the following assumptions:

1 The airflow is two-dimensional on XY plane, and the fibers are assumed to lie on the drafter symmetry plane, indicated with the white arrow on the centerline of Figure 1b.
2 The thin fibers are not modeled and do not affect the airflow.
3 Negligible heat transfer between fibers and the air.
4 The air is assumed to be an ideal gas with constant properties. Thus, despite slight variations in temperature and density, the viscosity value is assumed to be constant.

We used the open-source package OpenFOAM^®^ [39] v7 and its tools, i.e., blockMesh, the Scotch decomposition library, Open MPI 4.0.1, and rhoSimpleFoam to model the airflow. We applied a second-order linear upwind difference scheme to minimize numerical diffusion, and turbulence was modeled using the *k*-ε model [40,41]. To maintain consistency among the simulations, the mesh properties, e.g., cell size and number (∼30,000 cells), were updated according to geometrical modifications for each design parameter. We ran all simulations on the Niagara supercomputer cluster of the Digital Research Alliance of Canada.

## 3. Results and Discussion

**Figure 3:**
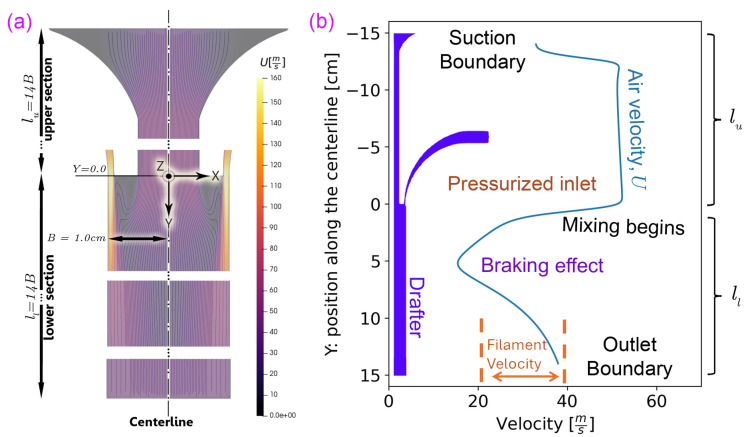
Simulation of the airflow within the base geometry. (**a**) The drafter velocity distribution and contours within the base geometry. The origin of the coordinate system is at the center of the drafter. (**b**) Centerline velocity for the base geometry. The velocity graph starts from the suction boundary $Y_{suction}=-14.0$ cm and extends to the outlet, i.e., $Y_{outlet}=+14.0$ cm. The braking effect corresponds to decelerating the flow by about $\frac{1}{2}$ to $\frac{1}{3}$. Typical fiber velocity is in the range of 20–40 $\frac{m}{s}$.

To begin, consider the airflow within the base geometry of the drafter in Figure 3a. The pressurized air exits the inlet vertically (at Y=0, the middle of the drafter), mixes slowly with the suction air along the lower section of the drafter $l_{l}$, and subsequently diffuses to the centerline until a reasonably uniform flow reaches the outlet.

We measured the velocity at different spots across the outlet (37–43 $\frac{m}{s}$) and across the suction (46–54 $\frac{m}{s}$). The measurements of the velocity at the boundaries validate the simulation results and confirm that the PP fibers have a minor influence on air velocity (Figure 3b). Note that inserting a manometer within the drafter would significantly disturb the air and fiber flows; therefore, the boundary measurements were considered sufficient. We believe the minor differences are due to the small pressure drop with PP, 3D effects, and experimental uncertainties.

**Figure 4:**
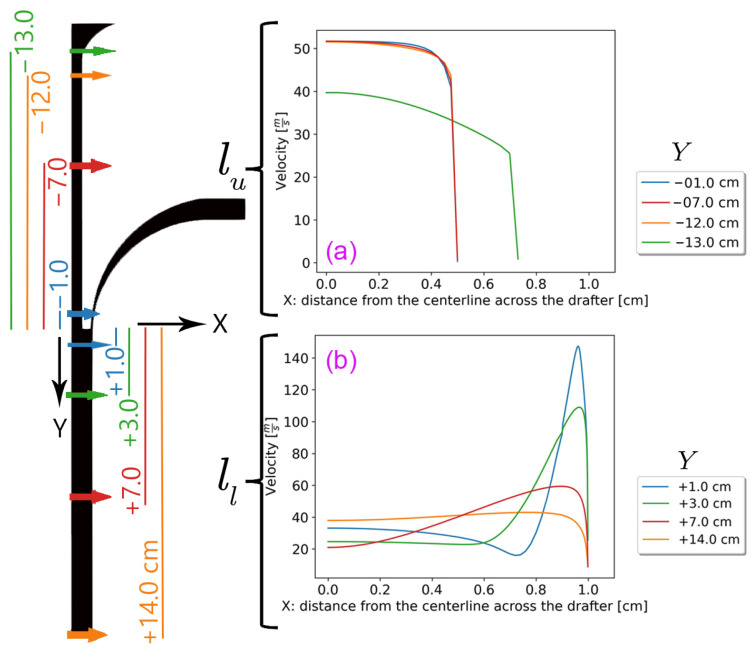
The velocity distribution across the drafter at different *Y* (**a**) the upper section along $l_{u}$, and (**b**) the lower section along $l_{l}$. The arrows and graphs corresponding to Y=±7 cm represent the middle points of each section. The average air velocity is more uniform in the upper section of the drafter.

The development of the predicted air velocity distribution (For consistency, the horizontal axis of all graphs in this study represents the horizontal location (*X*) and the vertical axis represents the vertical location (*Y*). Therefore, the plotted variable (e.g., *U*) may appear on either the horizontal or vertical axis) within the drafter is shown in Figure 3 and Figure 4. Figure 3b depicts the air velocity along the centerline of the base geometry. The velocity *U* is initially high at the upper section (Y<0) but decreases in the lower section (Y>0) as the drafter expands. It can be seen that the majority of the shear drag is exerted in the upper section, where the airflow is faster and more uniform. Notably, the fibers are below the freezing point in this area, and $U_{\mathrm{fiber}}$ remains constant, ranging between 20 and $40\;\frac{m}{s}$. We estimated this range with the continuity equation, knowing the melt flow rate, the die diameter, and the final fiber diameter. Importantly, within the lower section of the drafter, $U<U_{\mathrm{fiber}}$ is possible, which would induce an abrupt pull-up force. This shift in the direction of the relative velocity would subject fibers to significant localized stress, increasing the potential for fiber breakage. Such undesirable deceleration could be harsh, resulting in non-uniform deposition [12] and potentially causing drafter clogging. We term this rapid slowdown as the *braking effect*, which we aim to mitigate. Additionally, there are two sudden speedup zones, one at the drafter suction entrance and one by the outlet, which may cause excessive stress and fiber breakage.

Figure 4 shows *U* across the drafter at different vertical locations, illustrating flow development at different locations that are comparable to $U_{\mathrm{fiber}}$.

It is noteworthy that while the stress field in the lower section of the drafter and fiber breakage in this section are of lesser concern—since non-woven material is deposited on the belt regardless of whether the fibers are intact or broken—the non-uniformity of the stress can propagate upward into the melt, leading to melt breakage above the drafter.

In what follows, we concentrate on different aspects of the drafter design (Table 1) in each subsection, providing commentary on the modifications resulting from simulating various scenarios. All other parameters are kept constant in each subsection.

### 3.1. Effects of Inlet Pressure (*p*) on the Drafter Airflow

**Figure 5:**
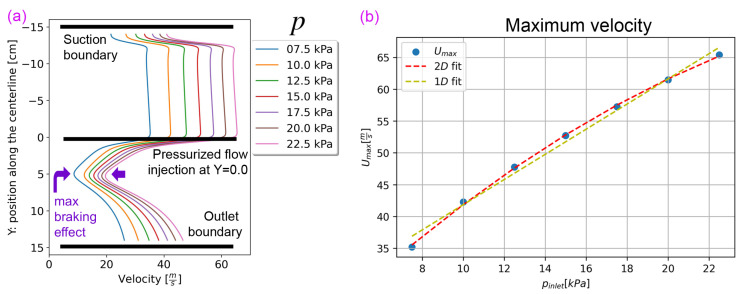
Effects of varying *p*. (**a**) *U* along the centerline versus *p*. The velocity curves have approximately a linear relation with the inlet pressure, and the location of the maximum braking effect does not depend on *p*. (**b**) Upper section maximum velocity versus *p*. Linear and quadratic curve fits are plotted, and the former is a good approximation.

In this section, we analyze the effects of the inlet pressure, *p*, with the three goals listed in Section 1. *p* is exerted via a fan or other pressurizer and provides the momentum for drawing. We ran airflow simulations with varying *p* to assess the extent of the shear drag and its uniformity. Figure 5 shows velocity distributions along the centerline, highlighting the extent of drawing achieved at different inlet pressures, and illustrates that the centerline velocity at any vertical position (*Y*) relative to *p* can be effectively modeled by a linear relationship within a wide range of pressures, as demonstrated in part b. This figure includes both linear and quadratic curve fits, with the linear fit proving to be a good approximation.

Tuning the drawing force is challenging and has traditionally been approached through trial and error. However, this insight assists in refining Equation (1), providing a practical method for estimating the net shear drag. This estimation assumes that the shear drag is primarily exerted in the upper section of the drawing region, where the air velocity is maximum ($U_{\max}$ in Figure 3b) and constant throughout the upper section. Therefore, shear drag is negligible anywhere else relatively.

The fiber velocity, $U_{\mathrm{fiber}}$, can be estimated based on the melt flow rate and the fiber’s final diameter.

(1) $$\begin{array}{ccc} \; & U_{max}=U_{\mathrm{upper}}\propto p & \mathrm{For}\;Y<0\\ \; & U_{\mathrm{fiber}}=a\;\mathrm{constant} & \mathrm{Estimated}\\ \; & F_{\mathrm{drag}}\approx F_{\mathrm{drag}}\left(U_{\max}\right)\propto {\left(U_{\max}-U_{\mathrm{fiber}}\right)}^{2} & \mathrm{Only}\;\mathrm{along}\;l_{u}\\ \; & \Rightarrow F_{\mathrm{drag}}\propto {\left(p-\mathrm{constant}\right)}^{2} & \mathrm{Net}\;\mathrm{shear}\;\mathrm{drag} \end{array}$$

It is worth noting that the constant of proportionality varies in the lower section of the drawing region. However, the shear drag force is significantly reduced in this region as the velocities *U* and $U_{\mathrm{fiber}}$ approach similar values.

Furthermore, changing the inlet pressure does not change the location of the maximum braking effect, as Figure 5a illustrates.

*p* has traditionally served as the primary means to control and regulate the air velocity and, consequently, the $F_{\mathrm{drag}}$. For our lab drafter, the practical range of pressure depends on the melt strength, and for our experimental setup, the effective range is 12.5–20 kPa. However, a noteworthy drawback of this control method is the alteration in flow pattern with increasing pressure, leading to significant velocity fluctuations and subsequent flow non-uniformity.

### 3.2. Effects of the Length at the Lower and Upper Sections ($l_{l}$ and $l_{u}$)

**Figure 6:**
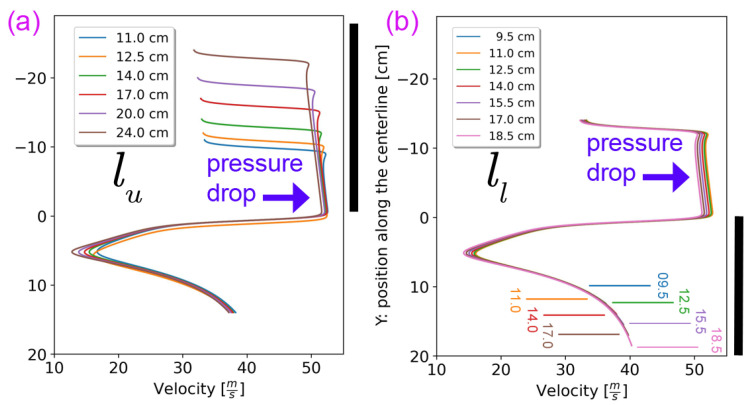
Distribution of *U* along the centerline of the drafter for different (**a**) $l_{u}$ and (**b**) $l_{l}$. Importantly, the longer the drafter, the higher the total drag time and the pressure drop.

The length parameters influence the flow development and mixing process as the pressurized air momentum is transferred to the centerline along the length of the drafter. We conducted simulations to assess the impact of varying the lengths of the upper ($l_{u}$) and lower ($l_{l}$) sections of the drafter, with base values of 14.0 cm for each. Figure 6a reveals that an increase in $l_{u}$ extends the segment with high *U*, and thus, increases the total $F_{\mathrm{drag}}$ time, enabling the drafter to exert greater drag force using the same inlet settings. Although the overall velocity profile remains essentially unchanged, a slight decrease is observed due to the pressure drop along the elongated drafter. Figure 6b demonstrates the potential to control $F_{\mathrm{drag}}$ by adjusting $l_{u}$, suggesting that an extensible drafter could provide significant benefits for precise control of the drawing force.

Similarly, as illustrated in Figure 6, the length of $l_{l}$ influences the extent of $F_{\mathrm{drag}}$. In part Figure 6b, the *Y* locations of endpoints of each drafter configuration are marked for visualization. The velocity profiles essentially exhibit the same overall shape, albeit with minor reductions due to pressure drops in longer ones. Therefore, although extending the lower section of the drafter perhaps enhances the drawing control, the proximity of the conveyor belt to the drafter outlet imposes practical limitations on extending $l_{l}$. Figure 6 also illustrates that modifying the values of $l_{l}$ and $l_{u}$ does not affect the braking effect. Additionally, changing the mixing length has a minimal impact on the mass flow rate due to minor pressure losses (Appendix A.2).

Therefore, we suggest the utilization of an extensible $l_{u}$ as a pragmatic approach for precise control of the drawing process. This strategy introduces a cost-effective alternative to simply increasing the inlet pressure. The adaptability afforded by an extensible $l_{u}$ enables post-adjustment following warm-up or material changes, thereby maximizing drawability without necessitating additional pressurized air, i.e., goals (a) and (c). Moreover, unlike variations in inlet pressure, adjustments to $l_{u}$ permit an increase in total shear drag force without compromising flow uniformity, goal (b).

### 3.3. Effects of Varying the Angles of the Side Walls ($t_{l}$, $t_{u}$, and $t_{uh}$) on the Drafter Airflow

We have come across studies that involve angled drafters (e.g., [14,33,42]) or drawing regions enclosed in angled walls, e.g., [43]. In this section, we analyze the drawing process within converging and diverging drafters, employing the slope indices denoting the positions of the sidewalls at the suction and the outlet, namely $t_{u}$ and $t_{l}$ indices, respectively. Figure 1d shows how these parameters yield distinct configurations. Furthermore, we explore a scenario where only the top half of the upper section is angled, denoted by $t_{uh}$. The rationale behind this arrangement will be detailed in Section 4, alongside pertinent considerations.

**Figure 7:**
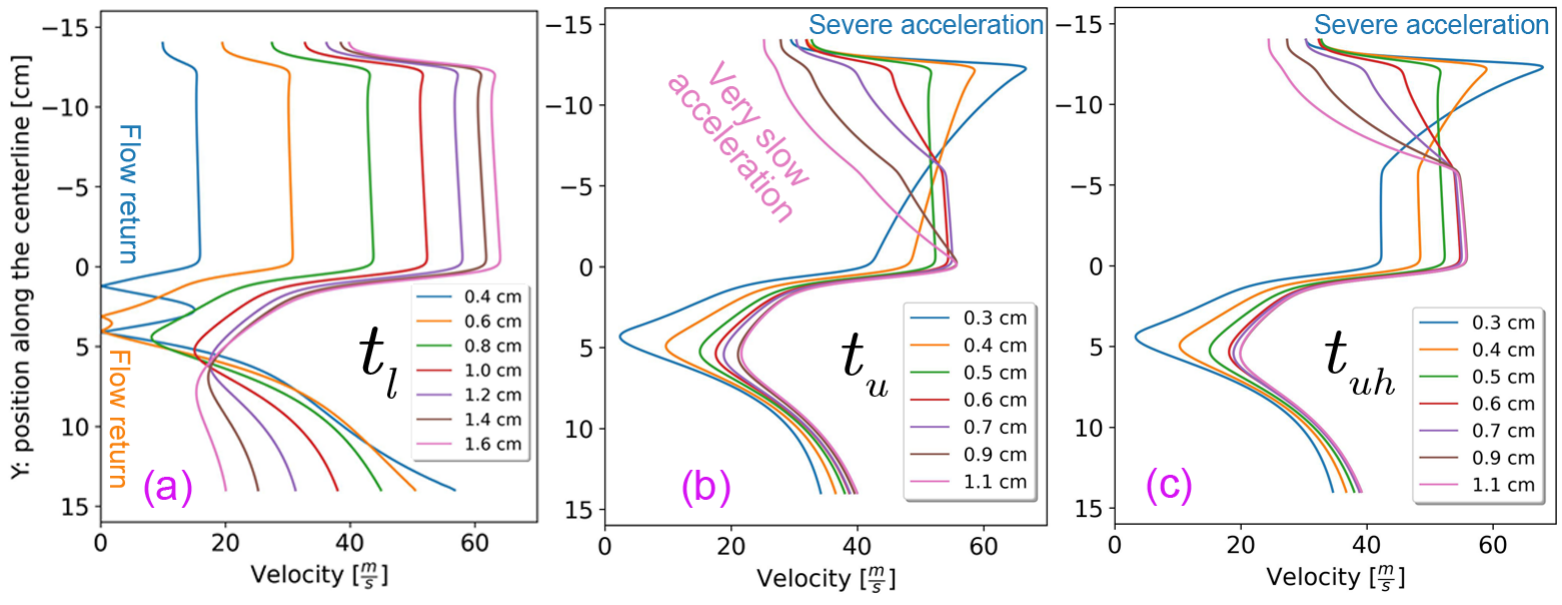
The centerline velocity distributions for different (**a**) $t_{l}$, (**b**) $t_{u}$, and (**c**) $t_{uh}$.

The values of $t_{l}$, $t_{u}$, and $t_{uh}$ are defined as the maximum horizontal width (*X*) from the centerline; for instance, the base values ($t_{l}$ = 1.0 cm, $t_{u}=0.5$, and $t_{uh}=0.5$) correspond to vertical walls; lower values mean the drafter end is leaning towards the centerline, and vice versa. Table 2 summarizes these values, and the corresponding centerline velocity distributions are illustrated in Figure 7.

Narrowing $t_{l}$ or $t_{u}$ disrupts flow uniformity, causing severe velocity changes that increase fiber breakage risk and may induce centerline backflow, compromising deposition uniformity.

On the other hand, as depicted in Figure 7, increasing both $t_{l}$ and $t_{u}$ can significantly enhance flow uniformity. This improvement addresses issues such as severe acceleration at the suction and the braking effect. However, there are limits to how much these values can be increased. For $l_{u}$ and $l_{uh}$, the average velocity and, consequently, total $F_{\mathrm{drag}}$ decrease along the upper section. For $l_{l}$, if the angle is too wide, the inlet airflow stays more on the side walls rather than moving toward the center of the drafter, resulting in poor momentum transfer from the pressurized air jet to the centerline. In other words, the high-speed air fails to reach the fibers properly before exiting the drafter, which is against the intended goal (c).

**Figure 8:**
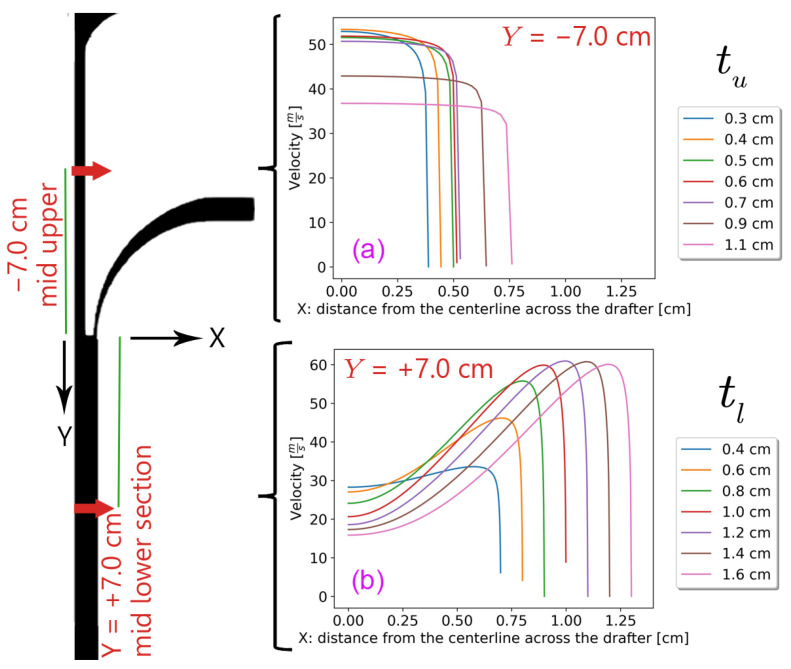
The velocity profiles across the drafter (**a**) at the middle of the upper section for different $t_{u}$ and (**b**) at the middle of the lower for different $t_{l}$.

**Figure 9:**
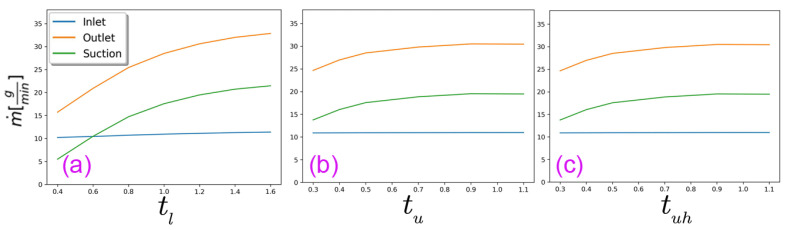
The mass flow rates at the boundaries for different (**a**) $t_{l}$, (**b**) $t_{u}$, and (**c**) $t_{uh}$.

Figure 8 illustrates velocity profiles at the middle of the lower and upper sections of the drafter for different $t_{u}$ and $t_{l}$, respectively. The former indicates a significant decrease in the average velocity at the centerline for high $t_{u}$, which is undesirable. The latter shows high velocities near the side wall, as the angle increases, which again is undesirable, indicating that wide angles work against our goal. The mass flow rates also confirm this upper limit since increasing the side wall angle does not result in additional air suction beyond a certain amount, as all graphs in Figure 9 plateau.

**Figure 10:**
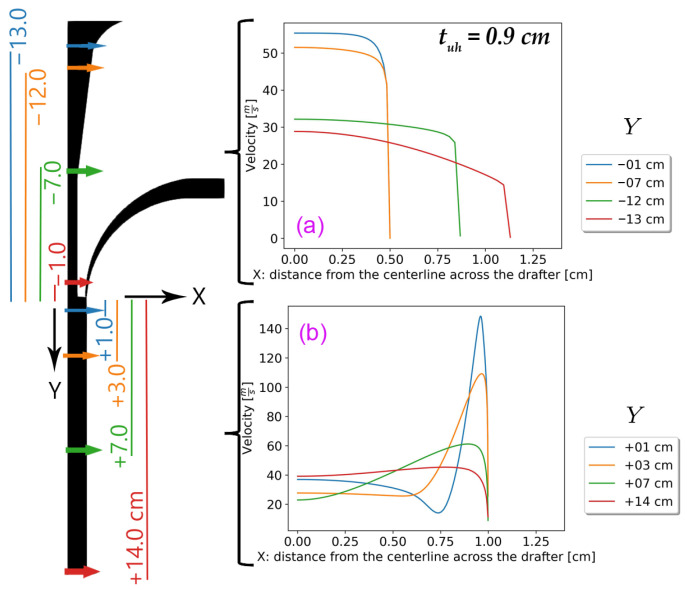
The velocity distribution with $t_{uh}=0.9$ cm across the mid-points of (**a**) the upper section and (**b**) the lower section.

Notably, it became apparent that we should add another variation to the analysis in this section. We varied $t_{uh}$ and simulated both low and high angles, as listed in Table 2. Figure 10 depicts the corresponding at different locations along the drafter for selected $t_{uh}=0.9$ cm. The profiles are comparable to those at the middle points of the upper and lower sections shown in Figure 8.

Based on our findings, we advocate for a slight positive angle for $t_{u}$ from our base geometry. Notably, increasing $t_{u}$ poses challenges in manufacturing an extendable angled $l_{u}$, and hence, we propose angling only the top half of the upper section, denoted as $t_{uh}$ = 0.5–0.6 cm, thereby facilitating the extension of the remaining half for precise control of $F_{\mathrm{drag}}$ and higher average *U*. Alternatively, we recommend $t_{u}$ = 0.5–0.7 cm if manufacturing an extensible $l_{u}$ and $t_{uh}$ proves to be exceedingly challenging. Finally, we recommend setting $t_{l}$ = 1.0–1.2 cm to effectively mitigate the braking effect and compensate for the slight reduction in shear drag force along the upper section resulting from changes in $t_{u}$. Additionally, we recommend setting $t_{l}$ = 1.0–1.2 cm to effectively mitigate the braking effect and compensate for the slight reduction in shear drag force along the upper section resulting from changes in $t_{u}$.

### 3.4. Effects of the Width Parameters (w, n and O) on the Drafter Airflow

**Figure 11:**
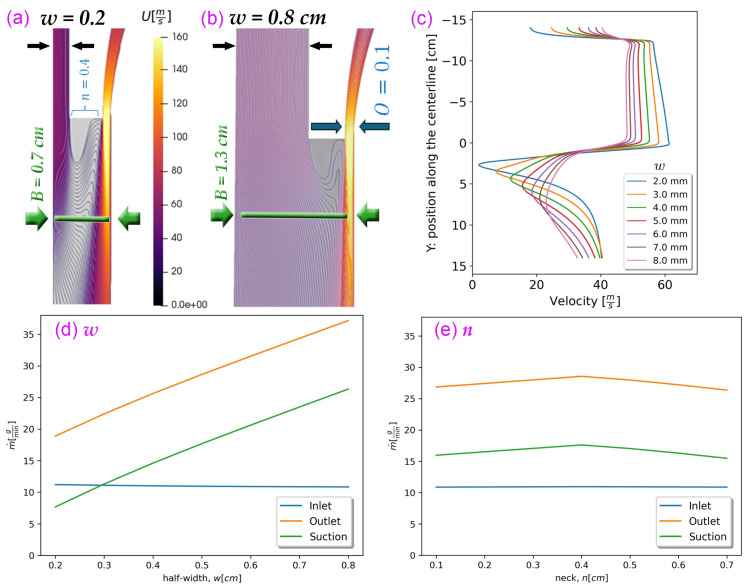
Parameters related to the width. (**a**) the lowest value of *w* and (**b**) its highest. (**c**) Distribution of *U* along the centerline for values of *w*. (**d**) Change in the boundary mass flow rates for different values of *w*. (**e**) Boundary mass flow rate, $\dot{m}$, versus neck, *n*.

Adjusting the width of the drafter corresponds to modifications in three variables, i.e., the suction width (*w*), the neck (*n*), and the orifice (*O*). Parts a and b of Figure 11 illustrate these variables and the velocity contours corresponding to the minimum and maximum values of *w*. These variables form the overall drafter breadth, B=w+n+O=1.0 cm in our base geometry. Here, *B* represents the overall distance from the centerline to the wall, *w* denotes the upper width, *n* is the gap separating the two streams, and *O* corresponds to the inlet orifice width.

Narrowing *w* intensifies mixing and gradients, forming a boundary layer between air streams in the lower section’s initial segment, while widening *w* shifts high-velocity flow toward the sidewall by expanding the mixing zone.

In our simulations, the parameter *w* varied from 0.2 to 0.8 cm, inducing a corresponding variation in *B* from 0.7 to 1.3 cm. As depicted in Figure 11c, at the low values of *w*, the velocities are elevated along the upper section at the expense of an intensified braking effect in the lower section, with the centerline air velocity potentially diminishing to zero around *Y* = 3 cm for exceedingly low *w*. Conversely, higher *w* values reduce the velocity variation span and decrease $F_{\mathrm{drag}}$ marginally along the upper section. This alteration significantly enhances uniformity within the drafter, compensating for the marginal loss in $F_{\mathrm{drag}}$. Plus, it mitigates the severe acceleration at the suction.

However, similar to the increase in $t_{l}$ and $t_{u}$, there is a limit to widening *w* as excessively high values delay the air mixing, causing pressurized air to exit the outlet without properly transferring its momentum to the centerline. Additionally, the average upper section centerline velocity decreases adversely, as shown in Figure 11c. As Figure 11d shows, increasing *w* increases the suctioned air for the same amount of inlet mass flow rate. More air that is not near the centerline is drawn in by a wider drafter, and the pressurized inlet energy is wasted because this air is too far from the centerline to exert a drag force.

Considering all these factors, qualitatively, the two velocity profiles corresponding to w=0.5 to 0.6 cm appear to yield a more uniform velocity distribution along the drafter, yet maintain the air momentum sufficiently close to the center. Expanding the drafter width through *w* yields effects similar to those observed with $t_{l}$, with both adjustments influenced by inlet energy consumption rate considerations. In both cases, an upper limit exists to ensure the minimization of excessive air at the suction point that does not contribute to shear drag on the fiber.

We considered changing *n* and maintained the values of *w* and *O* constant while varying *n*. Our simulations revealed that neither an increase nor a decrease in this parameter improved the airflow. Remarkably, our base geometry exhibits the most favorable performance with respect to the mass flow rates, as confirmed by Figure 11e. For low values of *n*, the velocity distributions in the mixing zone become more severe, while for high values, the high-speed inlet remains undesirably close to the side wall.

In conclusion, we propose the following values: maintaining our base values for n=0.4 and O=0.1 cm while slightly increasing *w* to the range of 0.5–0.6 cm (*B* = 1.0 to 1.2 cm) to enhance air velocity uniformity.

### 3.5. The Enhanced Design

**Figure 12:**
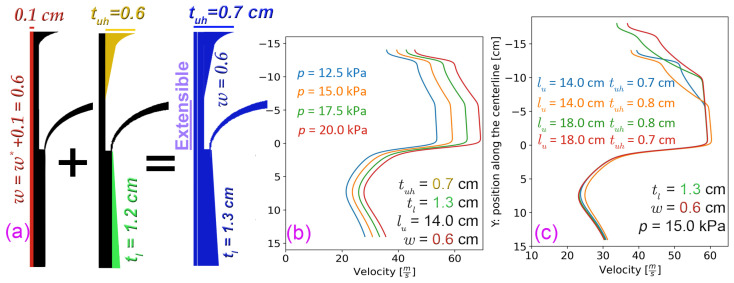
Centerline velocity subsequent to implementing the recommended values for the designed parameters. (**a**) The suggested value for *w* = 0.6 cm results in a shift for the suggested values of $t_{l}=1.2+0.1$ and $t_{uh}=0.1+0.6=0.7$. (**b**) Controlling *U* with *p* for the prioritized suggestion of $t_{uh}$ = 0.7 cm. (**c**) Precise control of *U* with an extensible upper section length $l_{u}$ for two recommended values of $t_{uh}$.

Thus far, we analyzed the variables listed in Table 1 for the three goals individually. As part of our analysis, we also examined the jet angle (see Figure 1c), and after comparing the simulation results, we found that changing the inlet angle does not contribute to the flow in terms of the goals we set. This section combines the outcomes and simulates the airflow within the new drafter design. We present the corresponding centerline velocity distribution in Figure 12, following the implementation of all recommendations outlined above. Figure 12a shows the new design schematics, and Figure 12b illustrates the impact of *p* on the velocity profile of the corresponding design, resulting in a shift of *U* towards the right or left.

With a very good linear approximation, this shift confirms that the drawing is proportionately scaled up or down after implementing our recommendations, and one can estimate the net drag force with Equation (1). In Figure 12c, the precise control of drawing is depicted with the extensible parameter $l_{u}$ at constant *p* to demonstrate the controllability of this variable. Additionally, two values of $t_{uh}=0.7$ and 0.8 cm that are within the recommended range are displayed for the centerline velocity. Nonetheless, since $t_{uh}=0.7$ offers a greater average *U*, it is advised that it be given priority.

### 3.6. Experimental Validation

**Figure 13:**
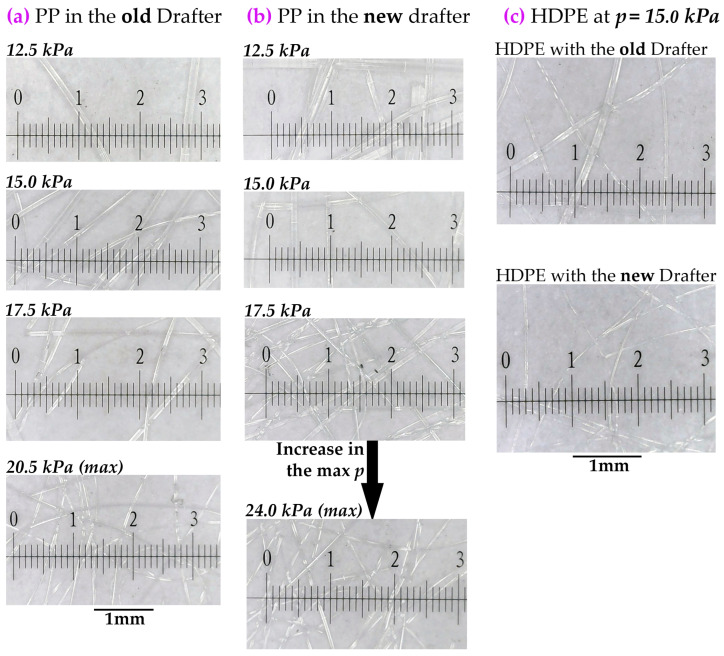
Comparative PP and HDPE fiber diameter measurements under an optical microscope using the old and new drafters. (**a**) PP fibers with the original drafter design at pressures up to 22.5kPa, at which breakage was observed. (**b**) PP fibers with the new drafter at a maximum pressure of 24.0kPa. (**c**) HDPE fibers with both drafter designs at 15.0kPa.

Based on the optimized values of the design parameters, a new drafter was constructed, integrated into our spunbonding machine, and evaluated through fiber sample collection. The finalized configuration incorporates $t_{uh}$ = 0.6 cm, $t_{l}$ = 1.2 cm, *w* = 0.6 cm, and an extensible $l_{u}$. Fiber diameters were measured after deposition, i.e., directly beneath the drafter and at a distance of 220 cm below the die. Figure 13 presents comparative results, showing that the new design consistently yields finer fibers at the same *p*, while also increasing the max *p* values without fiber breakage. These findings confirm that the proposed design modifications effectively align with our goals (c) and (a), respectively.

The newly built extensible length was set to $l_{uh}$ = 18.0 cm, and the quantitative fiber characteristics and maximum pressure measurements are presented in Table 3. Notably, significant improvements were achieved, including at least a 17.1% increase in the maximum operational pressure without fiber breakage and a 20.0% enhancement in fiber thinning performance.

**Figure 14:**
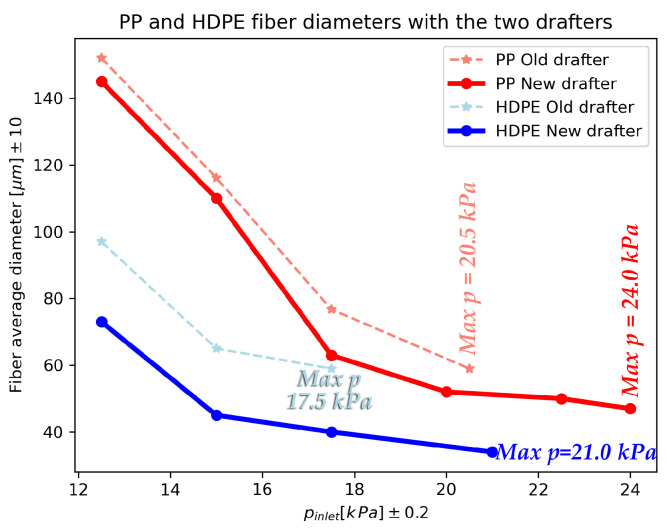
Comparison of PP and HDPE fiber diameters at different *p* values for the two drafter designs. Solid lines correspond to results obtained with the new drafter, while dashed lines represent the original design.

Furthermore, the fiber thinning achieved with the new design at different values of *p* is illustrated in Figure 14, demonstrating the overall improvements obtained through CFD-based design optimization and airflow simulation data for both polymer types. The solid lines indicate the new drafter, where the fibers have considerably thinner diameters and can be spun at higher *p*.

A few additional key observations are summarized below:

- The new design yielded a greater relative improvement for HDPE, which has inherently lower spinnability, underscoring the importance of flow uniformity in spunbonding, and thus achieving goal (b) with the new design.
- Reference pressures of 17.5kPa for PP and 15.0kPa for HDPE were selected in Table 3, as the corresponding microscope images in Figure 13 clearly illustrate meaningful reductions in fiber diameter for qualitative comparison.
- A linear correlation between *p* and fibers average diameter is apparent only in the right-hand portions of the graphs in Figure 14. At lower *p* values, gravity dominates the drawing process, whereas at higher *p* values the drag force of the drafter prevails, resulting in a more linear trend on the right side.

Overall, the experimental validation confirmed that the revised drafter design enables the production of finer fibers from both polymers while sustaining higher inlet pressures. Importantly, finer average diameters were achieved at identical pressure settings, demonstrating enhanced airflow drawing efficiency. Furthermore, the successful fabrication of finer fibers without breakage indicates a reduced risk of fiber failure with the new design. Additional experimental and manufacturing details are provided in Appendix C.

## 4. Conclusions and Recommendations

Various design parameters of a drafter for spunbonding (Table 1) were altered using CFD simulations. We altered the parameters after validating our air simulation velocity data on the base geometry in our lab. The alterations were applied gradually on the simulated design and were assessed based on the three goals in Section 1.

We observed a linear relationship between *p* and $U_{max}$, facilitating the calibration of the net $F_{\mathrm{drag}}$ using Equation (1). *p* has been traditionally used for drawing control, and we suggest the addition of an extensible $l_{u}$ to add precision to this method without disrupting the flow. Following that, we discussed that extending the length of either end of the drafter, i.e., $l_{u}$ or $l_{l}$, does not significantly alter the overall shape of *U*. However, it does impact the length of the profile and consequently affects the magnitude of the net $F_{\mathrm{drag}}$. Our investigation reveals that a notable enhancement in flow uniformity can be attained by angling the side walls of both the $t_{u}$ and $t_{l}$ away from the centerline of the drafter. Hence, we suggest $t_{uh}$ = 0.5–0.6 cm alongside the extendable angled $l_{u}$ (Figure 12). If that poses too many manufacturing challenges, we alternatively recommend $t_{u}$ = 0.5–0.7 cm. Additionally, we recommend setting $t_{l}$ = 1.0–1.2 cm for the lower section. Lastly, adjusting the width of the drafter corresponds to modifications in three variables. We propose maintaining our base values for n=0.4 and *O* = 0.1 cm while slightly increasing *w* to the range of 0.5–0.6 cm to enhance air velocity uniformity. Section 3.5 and Section 3.6 demonstrate the centerline airflow within the new drafter design after applying all the recommendations above and the experimental validation of the outcome performance with PP and HDPE fibers. Implementing the proposed modifications proved straightforward, while the experimental results demonstrated a substantial overall reduction in average fiber diameter.

For this analysis, the focus was restricted to the airflow dynamics within the drafter. The investigation was subsequently extended to the construction of the drafter and the comparative evaluation of average nonwoven fiber diameters. Experiments conducted on our spunbonding machine demonstrated that the new design, despite its simple modification, is significantly more efficient in achieving three objectives: producing finer PP and HDPE fibers at the same inlet pressure, enabling the formation of finer fibers at higher pressures, and accomplishing these improvements without fiber breakage.

While we acknowledge that industrial-scale setups may differ significantly in size, and direct generalization of our findings to larger drafters may not be straightforward, we believe our conclusions offer valuable insights. For instance, one potential method for scaling up a drafter involves extending its length along the *Z* axis, as depicted in Figure 2. Notably, our findings derived from the *X*-*Y* two-dimensional output remain applicable in such cases. The insights gained from this study can enhance the utility of alternative designs by improving the understanding of flow behavior across different scales (see Appendix B).

## Figures and Tables

**Table 1:** The design parameter space for the simulations. The starred (*) values correspond to the base geometry.

Parameter	Unit	Min			Range			Max
*p*	kPa	7.5	10	12.5	15.0 *	17.5	20	22.5
$l_{l}$	cm	9.5	11.0	12.5	14.0 *	15.5	17.0	18.5
$l_{u}$	cm	11.0	12.5	14.0 *	17.0	20.0	24.0	Extensible
${t_{l}}^{†}$	cm	0.4	0.6	0.8	1.0 *	1.2	1.4	1.6
${t_{u}}^{†}$	cm	0.3	0.4	0.5 *	0.6	0.7	0.9	1.1
${t_{uh}}^{†}$	cm	0.3	0.4	0.5 *	0.6	0.7	0.9	1.1
*w*	cm	0.2	0.3	0.4	0.5 *	0.6	0.7	0.8

^†^ The design parameter corresponds to a wall angle. For clarity in the repository mesh, the end-point locations are listed in centimeters (cm), while the corresponding angles are provided in the following sections.

**Table 2:** Selected values of $t_{l}$, $t_{u}$, and $t_{uh}$ with the corresponding slopes. The starred (*) values correspond to the base geometry. The slopes for $t_{uh}$ are higher than for $t_{u}$ because of the shorter length.

Parameter	Min						Max
** $t_{l}$ **	0.4 cm	0.6	0.8	1.0 *	1.2	1.4	1.6
$t_{l}$ slope ^†^	$\frac{-3}{70}\frac{\mathrm{mm}}{\mathrm{mm}}$	$\frac{-2}{70}$	$\frac{-1}{70}$	0 *	$\frac{+1}{70}$	$\frac{+2}{70}$	$\frac{+3}{70}$
** $t_{u}$ **	0.3	0.4	0.5 *	0.6	0.7	0.9	1.1
$t_{u}$ slope ^†^	$\frac{-2}{125}$	$\frac{-1}{125}$	0 *	$\frac{+1}{125}$	$\frac{+2}{125}$	$\frac{+4}{125}$	$\frac{+6}{125}$
** $t_{uh}$ **	0.3	0.4	0.5 *	0.6	0.7	0.9	1.1
$t_{uh}$ slope ^†^	$\frac{-2}{70}$	$\frac{-1}{70}$	0 *	$\frac{+1}{70}$	$\frac{+2}{70}$	$\frac{+4}{70}$	$\frac{+6}{70}$

^†^ The resulting slope of the corresponding wall in the row above.

**Table 3:** The comparative results of polypropylene and high-density polypropylene with the new drafter design in spunbonding for validation of the simulation outcome.

Criteria	PP Fiber Average Average at 17.5 kPa	Max Operating *p* Without Breakage	HDPE Fiber Average Average at 15.0 kPa	Max Operating *p* Without Breakage
Old drafter	76.7 μm	20.5 kPa	65.0 μm	17.5 kPa
New drafter	63.1 μm	24.0 kPa	45.2 μm	21.0 kPa
Improvement	21.6%	17.1%	43.8%	20.0%

**Figure A1:**
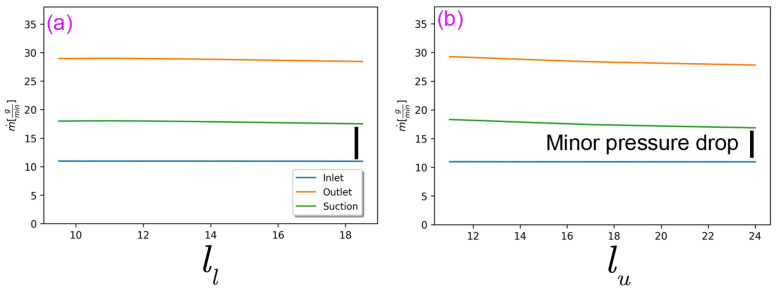
Mass flow rates versus different values of (**a**) $l_{l}$ and (**b**) $l_{u}$.

**Figure A2:**
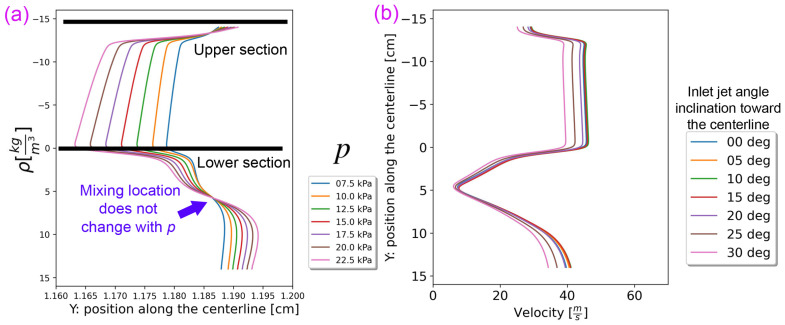
Additional results of the simulations. (**a**) The air density along the centerline for different values of *p* shows that, first, the flow can be assumed incompressible, and second, the location of mixing does not change with *p*. (**b**) Centerline velocity for different jet angles: $0^{\circ }$ means parallel to the centerline, and other degrees lean into the centerline.

**Figure A3:**
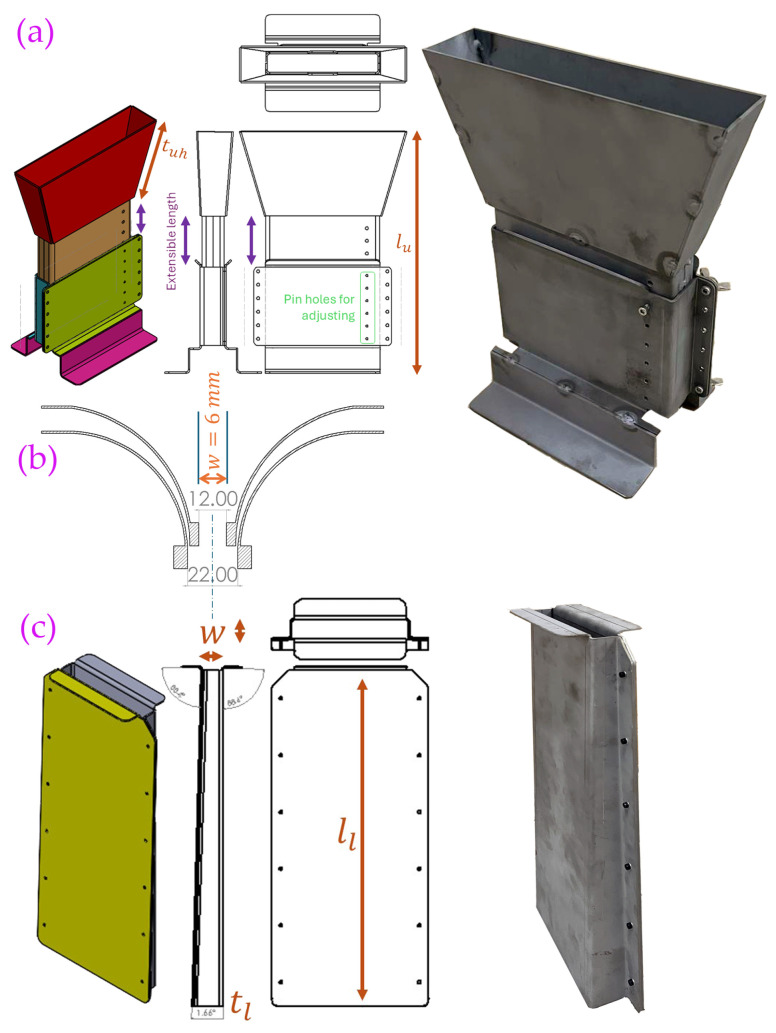
The drafter CAD files on Solidworks and pictures after building. (**a**) The top part was built separately with an extensible $l_{u}$ which adjusts a pin. It was installed in the middle part. (**b**) The middle part was a part installed as a part of the spunbonding machine, where we merely extended *w* by moving the air channels further from each other. No new part was necessary for this section. (**c**) The new lower section.

**Figure A4:**
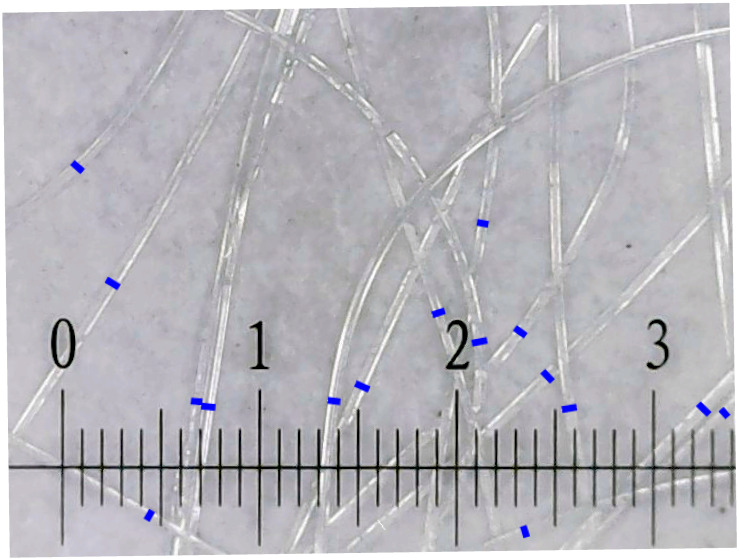
Example 1600× micrograph and ImageJ measurement of HDPE fibers at *p* = 12.5 kPa within the new drafter.

## Data Availability

The original data presented in the study are openly available in Materials Cloud at https://doi.org/10.24435/materialscloud:54-2z at reference [44].

## Appendix A. Additional Information of the Simulation

### Appendix A.1. Using the Repository for Replication

Two OpenFOAM are available at [44], corresponding to the base and final geometry after applying all recommendations. The blockMeshDict includes the design parameters and can be edited manually. The repository is cited within the manuscript and contains these cases. The 0/p files can be adjusted for further investigations. One should note that modifying these cases requires minimal skills on OpenFOAM-v7 in building the mesh and running the cases

1 blockMesh
2 mapFields using the base geometry
3 MPI library for parallel running with decomposePar
4 rhoSimpleFoam

with the given order. Additionally, we provide some CFD information of the cases in the repository in Table A1.

**Table A1:** CFD conditions and critical boundaries.

Velocity Boundary Conditions		Comment
inlet	pressureInletVelocity	Pressure-driven
suction	pressureInletVelocity	Only inlet
outlet	pressureInletOutletVelocity	Only outlet
**Pressure Boundary Conditions**		**Allows Testing Compressibility**
inlet	totalPressure	*p*
suction	fixedValue	Ambient
outlet	fixedValue	Ambient
**CFD Assumptions**		
Ideal gas	Two-dimensional	Constant air viscosity

### Appendix A.2. Additional Results

In this section, we provide three additional figures generated during this study. Figure A1 shows that the extensions of the length parameters results in minor pressure/mass flow rate drop, and thus, they are suitable for tuning of the drawing force.

Notably, the “braking effect” was assessed qualitatively by systematically varying the drafter geometry and observing the resulting trends in the velocity profile. The effect originates from the drafter geometry, while pressure variations primarily alter its intensity rather than its location (see Figure 5a and Figure A2a), which provides insights into fiber drawability and potential breakage.

## Appendix B. Dimensionless Forms

In this section, we present the takeaways in dimensionless forms to enable the generalization of this study for various drafter sizes. This approach provides an overview of our recommendations for the sizing and calibrating pressure beyond our base geometry. Additionally, the results can be scaled up for industrial applications. We introduce the dimensionless forms of the design variables (marked with ∼) and list them with the corresponding base and suggested values in Table A2. Notably, Equation (A1) demonstrates the relationship with the parameters correlating with the drafter width.

(A1) $$w+n+O=B\Rightarrow \frac{w}{B}+\frac{n}{B}+\frac{O}{B}=\frac{B}{B}=\tilde{w}+\tilde{n}+\tilde{O}=\tilde{B}=1$$

**Table A2:** The base and recommended design parameters with the corresponding dimensionless forms and values.

Parameter	Base [Unit]	Dimensionless	$\tilde{\mathrm{Base}}$	Recommended
*p*	15.0 kPa	$\tilde{p}=\frac{p\rho B^{2}}{\mu^{2}}\times 10^{9\;†}$	8.29	5.52–10.10
$l_{l}$	14.0 cm	$\tilde{l_{l}}=\frac{l}{B}$	14.0	14.0
$l_{u}$	14.0 cm	$\tilde{l_{u}}=\frac{l_{u}}{B}$	14.0	Extensible
$t_{l}$	1.0 cm	${\tilde{t}}_{l}=\frac{t_{l}}{B}$	1.0	1.0–1.2
${t_{u}}^{‡}$	0.5 cm	${\tilde{t}}_{u}=\frac{t_{u}}{B}$	0.5	0.6–0.7
${t_{uh}}^{‡}$	0.5 cm	${\tilde{t}}_{uh}=\frac{t_{uh}}{B}$	0.5	0.6–0.7
*w*	0.5 cm	$\tilde{w}=\frac{w}{B}$	0.5	0.54–0.55
*n*	0.4 cm	$\tilde{n}=\frac{n}{B}$	0.4	0.4
*O*	0.1 cm	$\tilde{O}=\frac{O}{B}$	0.1	0.1

^†^ *μ* is the air viscosity, 1.81 × 10^−5^ Pa·s. ^‡^ Either *t_u_* or *t_uh_* can be applied.

## Appendix C. Additional Experimentation Details

The three-dimensional models of the upper and lower components were developed using SolidWorks Education Edition 2025 (Dassault Systèmes, Vélizy-Villacoublay, France). The drafter was fabricated from cold-rolled steel with a thickness of 1.897mm, employing sheet metal processing, M3 screws and bolts, welding, and silicone sealant to ensure proper sealing of joints and gaps. The extensible length of the upper section was adjusted via pin holes located on both sides.

The middle section was mounted onto the spunbonding machine, and the inlet positions were aligned by adjusting the air ducts. Figure A3 presents the CAD model alongside the new parts that we built and installed.

Lastly, Figure A4 shows a representative micrograph acquired using a Wendry Digital Electron Microscope optical microscope and analyzed with ImageJ [38]. Spunbonded fiber samples were examined under 1600× magnification without any imaging filter. Each fiber diameter was manually marked (blue lines in Figure A4) and calibrated using a RM Taicols Micrometer Sliding Optical Glass Measuring Grid. ImageJ then exported the measurements in .csv format, and the fiber diameters for each batch were averaged.

## References

[1] Shealy O. (1965). Spunbonded products—A new concept in utilization of fibrous materials. Text. Res. J..

[2] Allison K.G. (1967). Process for Forming Non-Woven Filamentary Structures from Fiber-Forming Synthetic Organic Polymers. U.S. Patent.

[3] Kase S., Matsuo T. (1965). Studies on melt spinning. I. Fundamental equations on the dynamics of melt spinning. J. Polym. Sci. Part A Polym. Chem..

[4] Bansal V., Shambaugh R.L. (1996). On-Line determination of density and crystallinity during melt spinning. Polym. Eng. Sci..

[5] Yarin A.L., Sinha-Ray S., Pourdeyhimi B. (2011). Meltblowing: Multiple polymer jets and fiber-size distribution and lay-down patterns. Polymer.

[6] Pu Y., Zheng J., Chen F., Long Y., Wu H., Li Q., Yu S., Wang X., Ning X. (2018). Preparation of Polypropylene Micro and Nanofibers by Electrostatic-Assisted Melt Blown and Their Application. Polymers.

[7] Sun G., Ruan Y., Wang X., Xin S., Chen Y., Hu W. (2019). Numerical Study of Melt-Blown Fibrous Web Uniformity Based on the Fiber Dynamics on a Collector. Ind. Eng. Chem. Res..

[8] Ericson C., Baxter J. (1973). Spunbonded nonwoven fabric studies: I: Characterization of filament arrangement in the web. Text. Res. J..

[9] Chen C.H., White J.L., Spruiell J.E., Goswami B.C. (1983). Dynamics, Air Drag, and Orientation Development in the Spunbonding Process for Nonwoven Fabrics. Text. Res. J..

[10] Rizvi A., Andalib Z.K., Park C.B. (2017). Fiber-spun polypropylene/polyethylene terephthalate microfibrillar composites with enhanced tensile and rheological properties and foaming ability. Polymer.

[11] Zhao C., Mark L.H., Alshrah M., Soltani I., Lee P.C., Park C.B. (2019). Challenge in manufacturing nanofibril composites with low matrix viscosity: Effects of matrix viscosity and fibril content. Eur. Polym. J..

[12] Vadood M., Semnani D. (2011). Optimization of fiber distribution in spunbond non-woven structure. Fibers Polym..

[13] Venu L.B.S., Shim E., Anantharamaiah N., Pourdeyhimi B. (2012). Three-Dimensional Structural Characterization of Nonwoven Fabrics. Microsc. Microanal..

[14] Kanai T., Kohri Y., Takebe T. (2018). Theoretical analysis of the spunbond process and its applications for polypropylenes. Adv. Polym. Technol..

[15] Sun G., Yang J., Xin S., Yu R., Wang X. (2018). Influence of Processing Conditions on the Basis Weight Uniformity of Melt-Blown Fibrous Webs: Numerical and Experimental Study. Ind. Eng. Chem. Res..

[16] Doufas A.K., Dairanieh I.S., McHugh A.J. (1999). A continuum model for flow-induced crystallization of polymer melts. J. Rheol..

[17] Doufas A.K., McHugh A.J., Miller C. (2000). Simulation of melt spinning including flow-induced crystallization Part I. Model development and predictions. J. Non-Newtonian Fluid Mech..

[18] Tanner R.I., Qi F. (2009). Stretching, shearing and solidification. Chem. Eng. Sci..

[19] Derakhshandeh M., Hatzikiriakos S.G. (2012). Flow-induced crystallization of high-density polyethylene: The effects of shear and uniaxial extension. Rheol. Acta.

[20] Rawal A., Mukhopadhyay S. (2014). Melt spinning of synthetic polymeric filaments. Advances in Filament Yarn Spinning of Textiles and Polymers.

[21] Embabi M., Kweon M.S., Chen Z., Lee P.C. (2020). Tunable tensile properties of polypropylene and polyethylene terephthalate fibrillar blends through micro-/nanolayered extrusion technology. Polymers.

[22] Wang H., Jin X., Mao N., Russell S.J. (2010). Differences in the Tensile Properties and Failure Mechanism of PP/PE Core/Sheath Bicomponent and PP Spunbond Fabrics in Uniaxial Conditions. Text. Res. J..

[23] Wagner M.H., Schulze V., Göttfert A. (1996). Rheotens-mastercurves and drawability of polymer melts. Polym. Eng. Sci..

[24] Wagner M.H., Collignon B., Verbeke J. (1996). Rheotens-mastercurves and elongational viscosity of polymer melts. Rheol. Acta.

[25] Midha V.K., Dakuri A. (2017). Spun bonding Technology and Fabric Properties: A Review. J. Text. Eng. Fash. Technol..

[26] Ziabicki A. (1961). Mechanical aspects of fibre spinning process in molten polymers—Part III.: Tensile force and stress. Kolloid-Zeitschrift.

[27] Majumdar B., Shambaugh R.L. (1990). Air drag on filaments in the melt blowing process. J. Rheol..

[28] Bansal V., Shambaugh R.L. (1998). On-line Determination of Diameter and Temperature during Melt Blowing of Polypropylene. Ind. Eng. Chem. Res..

[29] Chen T., Wang X., Huang X. (2004). Modeling the Air-Jet Flow Field of a Dual Slot Die in the Melt Blowing Nonwoven Process. Text. Res. J..

[30] Xin S., Wang X. (2012). Shear flow of molten polymer in melt blowing. Polym. Eng. Sci..

[31] Bo Z. (2014). Experimental and Numerical Simulation Study of an Air drawing Model of Polyethylene Terephthalate (PET) Polymer and Model of Air Jet Flow Field in the Spunbonding Nonwoven Process. Fibres Text. East. Eur..

[32] Tabatabaei A., Barzegari M.R., Mark L.H., Park C.B. (2017). Visualization of polypropylene’s strain-induced crystallization under the influence of supercritical CO_2_ in extrusion. Polymer.

[33] Chen K., Ghosal A., Yarin A.L., Pourdeyhimi B. (2020). Modeling of spunbond formation process of polymer nonwovens. Polymer.

[34] Ataei M., Bussmann M., Shaayegan V., Costa F., Han S., Park C.B. (2021). NPLIC: A machine learning approach to piecewise linear interface construction. Comput. Fluids..

[35] Guapacha J., Barbosa J., Vallés E.M., Quinzani L.M., Failla M.D. (2020). Improving melt strength of polypropylene by minimal branching and blending. J. Appl. Polym. Sci..

[36] Liang J.Z. (2020). Melt strength and stretching ratio of low-density polyethylene composites loaded with nanoscale zinc oxide. Adv. Ind. Eng. Polym. Res..

[37] Abu Naser S. (2019). In-Situ Nano Fibrillation of Polyethylene Terephthalate (PET) in Polypropylene (PP) Through Spunbond Process. Master’s Thesis.

[38] Schindelin J., Arganda-Carreras I., Frise E., Kaynig V., Longair M., Pietzsch T., Preibisch S., Rueden C., Saalfeld S., Schmid B. (2012). Fiji: An open-source platform for biological-image analysis. Nat. Methods.

[39] Weller H.G., Tabor G., Jasak H., Fureby C. (1998). A tensorial approach to computational continuum mechanics using object-oriented techniques. Comput. Phys..

[40] Ignat L., Pelletier D., Ilinca F. (2000). A universal formulation of two-equation models for adaptive computation of turbulent flows. Comput. Methods Appl. Mech. Eng..

[41] Sinha K. (2001). Analysis of the k-Epsilon Turbulence Model for Simulation of Compressible Flows. Ph.D. Thesis.

[42] Bhat G.S., Malkan S.R. (2001). Extruded continuous filament nonwovens: Advances in scientific aspects. J. Appl. Polym. Sci..

[43] Beyreuther R., Brünig H. (2006). Dynamics of Fibre Formation and Processing: Modelling and Application in Fibre and Textile Industry.

[44] Mohajer B., Park C.B., Bussmann M. (2025). Computational optimization of a drafter for spunbonding polymeric filaments. arXiv.

